# The herd immunity attractor: selection, drift, and the steady-state undersupply of vaccination in 195 countries, 1980–2025

**DOI:** 10.3389/fpubh.2026.1965676

**Published:** 2026-10-22

**Authors:** Aras Yolusever

**Affiliations:** Department of Economics, Faculty of Economics and Administrative Sciences, Istanbul Kültür University, Istanbul, Türkiye

**Keywords:** evolutionary game theory, health systems, herd immunity, immunization policy, positive externality, replicator-mutator dynamics, vaccination coverage, WUENIC

## Abstract

**Introduction:**

Vaccination is the canonical positive externality in health economics: because each vaccination protects others, decentralized choice is predicted to stall below the herd immunity threshold. Yet little evidence exists on the law of motion that governs national coverage. This study estimates it directly.

**Methods:**

I model the vaccinate-or-not decision of a heterogeneous household population as an expected-utility problem whose net payoff declines in the vaccinated share, aggregate it through an explicit revision protocol into a selection-mutation (replicator-mutator) equation in which payoff-responsive switching coexists with health-system contact and attrition hazards, and estimate the resulting equation on the World Health Organization/United Nations Children's Fund (WHO/UNICEF) Estimates of National Immunization Coverage (WUENIC, 2025 revision) for 195 countries over 1980-2025, with UN cohort denominators and World Bank income and health-system capacity controls. An exact identification result shows that the linear turnover coefficient equals the difference between the law of motion at full and at zero coverage, so it is identified from boundary observations independently of the payoff parameters, whereas the payoff slope is identified only where coverage traverses the simplex.

**Results:**

First, in the interior of the coverage range, the dynamics are dominated by a strong linear restoring force, implying an annual turnover of 15%–25% of the relevant pool; the payoff slope is statistically weak there, and interior data cannot distinguish institutional flows from payoff-insensitive revision, so the estimated hazards bound the institutional component from above. Second, the free-riding signature appears where it is identified: sharply in the Hib rollout, which spans the full coverage range, and weakly on the first-dose DTP margin. Third, every country's law of motion has a stable interior rest point; the births-weighted world rest point for first-dose measles vaccination is 78%, below the 92%–94% herd band; 74% of countries rest below it, and a coronavirus disease 2019 (COVID-19) era shift lowered the median rest point by 3.3 points.

**Discussion:**

Because the leverage of retention over payoff instruments equals 1/(1 − x^*^) under the replicator protocol and diverges along the last mile under any smooth, bounded choice protocol, closing the gap is a default-and-retention problem rather than a price problem.

## 1 Introduction

Vaccination holds a special place in health economics because it is the clearest large-scale example of a positive consumption externality. A vaccinated individual protects not only herself but also everyone she would otherwise have infected. Hence, the private benefit of vaccination falls short of the social benefit, and the gap widens as coverage rises. When most of the community is immune, the residual infection risk that motivates the marginal household is small, whereas the social value of pushing coverage across the herd immunity threshold remains large. The theoretical implication has been understood at least since Fine and Clarkson (1) and was formalized in the public economics literature by Brito et al. (2), Francis (3), and Geoffard and Philipson (4), and in the game-theoretic epidemiology literature by Bauch and Earn (5), Galvani et al. (6), and Reluga and Galvani (7): decentralized vaccination choice has a Nash equilibrium strictly below the social optimum, and elimination of a disease is not an equilibrium of voluntary behavior.

The discipline lacks direct evidence on the mechanism behind this prediction. The theoretical literature is almost entirely analytical or simulation-based (8, 9); the empirical vaccination literature, for its part, estimates levels, determinants, and quasi-experimental responses in coverage, but it does not estimate the dynamical system that the theory specifies. This is a substantive omission, not a technical one. The externality argument is a statement about dynamics: it says that the annual change in coverage should respond to the level of coverage with a particular sign, that the response should operate through the mass of households who can still switch, and that the resulting flow should vanish at an interior rest point below the herd threshold. Each claim is testable on the world's largest coverage panel, yet none has been tested.

This study performs that test. The empirical object is the WHO/UNICEF Estimates of National Immunization Coverage (WUENIC, 2025 revision), an annual country-by-antigen panel of the vaccinated share of each birth cohort covering 195 countries from 1980 to 2025 (10). The vaccinated share of a cohort is a genuine two-state population share: it lives on the unit simplex, it sums with the unvaccinated share to one, and its year-to-year movement is exactly the object that an evolutionary law of motion describes. I derive the law of motion from microeconomic primitives in two steps. First, a household chooses whether to complete a vaccination schedule by comparing the expected utility loss from infection, which declines with aggregate coverage because of herd protection, with the full cost of vaccinating, which includes money and time prices as well as the psychological cost documented in the vaccine-hesitancy literature (11, 12). Second, the population does not re-optimize continuously. In any year, only a fraction of households actively revise their choice in response to the payoff difference; the remainder is carried by two payoff-blind flows, an institutional enrollment hazard that draws unvaccinated children into the schedule whenever the household touches the health system, and an attrition hazard that removes vaccinated-track children through migration, provider discontinuity, misinformation shocks, and administrative failure. The resulting dynamic is a replicator-mutator equation (13–16), in which the selection term captures the economics of free riding, and the mutation terms carry the institutions.

The relationship between the household model and the aggregate equation is made explicit in Sections 2.2 and 2.3. Households are heterogeneous in vaccination cost; revision opportunities arrive at a finite rate; a revising household imitates the better-performing choice with a probability proportional to the payoff difference; and, independently of any payoff comparison, an unvaccinated-track child meets the health system with a contact probability and is enrolled with a conversion probability, while a vaccinated-track household drops out with an attrition probability. The mean-field limit of this process is the replicator-mutator equation, and the two payoff-blind hazards are health-system quantities: the contact probability depends on the reach of the workforce and primary-care facilities, the conversion probability on supply reliability and default scheduling, and the attrition probability on continuity of care and confidence shocks. The framework of health-system building blocks, governance, financing, workforce, medical products, information, and service delivery (17), and the recent panel evidence that workforce density, public financing, and governance quality are the leading determinants of service coverage in low- and middle-income countries (18–20) therefore enter the model at a precise place: they determine the parameters of the mutation channel, not those of the payoff channel. This is why the article's central finding, that mature coverage dynamics are dominated by the linear flows, is a statement about health systems rather than about household indifference.

The structure of this equation defines the identification strategy. The selection term is multiplied by *x*(1 − *x*), the variance of the two-state population and therefore vanishes at both boundaries of the simplex; the mutation flows are linear in *x* and remain active at the boundaries. Section 2.6 states this as an exact result: the four reduced-form coefficients are a one-to-one reparametrization of a cubic in lagged coverage, and the turnover coefficient equals the difference between the law of motion's values at full and at zero coverage, two quantities that involve no payoff parameter. Boundary and near-boundary observations therefore identify mutation hazards separately from selection, a functional-form identification that requires no instrument but does require the affine payoff structure of the model. Conversely, in the narrow interior window where 83% of the panel lives, the level and the slope of the payoff difference are nearly collinear (the sample correlation of the two selection regressors is 0.87), so the frequency-dependence slope, which is the free-riding signature, is weakly identified there and must be sought where the data traverse the simplex: in new-vaccine rollouts.

Three findings follow. First, the interior dynamics of established antigens are dominated by the linear turnover channel. For first-dose measles vaccination (MCV1), the linear restoring coefficient is −0.248 with a *t*-statistic above 10 under two-way fixed effects. The point estimate decomposes, for the median country, into an enrollment hazard of about 0.20 and an attrition hazard of about 0.045 per year, and a measurement-robust dating places the joint turnover between 0.15 and 0.25. The selection terms are statistically weak in the interior. I also show, however, that the reduced form cannot, on interior data alone, distinguish payoff-blind institutional flows from a payoff-responsive revision protocol whose switching probability is locally flat in coverage: under a best-response protocol the revision rate itself appears as a linear restoring term. The estimated hazards are therefore upper bounds on the institutional component, and the phrase “payoff-blind” is used throughout in the operational sense of payoff-insensitive at the margin of the observed coverage window. The rollout antigens, where the variance-weighted form fits, are what discriminate between the two readings. Coverage behaves less like a running Nash computation and more like a convention maintained by an institutional default and eroded by drift. Second, the free-riding signature appears exactly where we identify it. In the Hib rollout, which traverses the whole coverage range within the sample, the payoff difference declines significantly in coverage and implies that selection alone would stall the rollout at 87%; the first-dose DTP margin, which measures first contact with the system rather than completion, shows a weaker version of the same declining payoff, without an interior stall. Third, every country's estimated law of motion has a stable interior rest point, reached with a half-life of about two years. The median rest point for MCV1 is 87.6%, and the births-weighted world rest point is 78.0%, against a measles herd immunity band of 92%–94% implied by a basic reproduction number of 12 to 18 (21); 74% of countries rest below the band. A post-2019 shift in the law of motion, in line with the pandemic-era evidence on vaccine confidence and service disruption (22–24) and located by the estimates in the enrollment channel, lowers the median rest point by a further 3.3 points.

The policy content is a closed-form leverage result. Because a payoff instrument (a subsidy, a conditional transfer, or an information campaign that raises perceived risk) enters the law of motion multiplied by *x*(1 − *x*), while a retention instrument (defaults, reminder systems, school-entry verification) enters multiplied by *x*, the ratio of their steady-state effects is 1/(1 − x^*^), which is about eight at the observed rest point and grows without bound as coverage approaches one. The result is not an artifact of the replicator form: under any revision protocol in which the probability of choosing vaccination is a smooth, bounded function of the payoff difference, the marginal effect of a payoff shift is proportional to the derivative of that probability, which must vanish as coverage approaches one, whereas the marginal effect of a retention shift does not. The same arithmetic reconciles two bodies of evidence that appear to conflict: incentives raise coverage strongly in low-coverage settings, where *x*(1 − *x*) is close to its ceiling, the coverage share itself, so that a payoff instrument has the same per-unit leverage as a retention instrument, and weakly in high-coverage settings, where it is far below that ceiling. Calibrated on the estimates, moving the median country from its rest point to the herd band requires a payoff shift about 12 times larger than the equivalent improvement in retention. The last mile of herd immunity is not a price problem; it is a default-and-retention problem.

Two further themes run through the article. The first concerns heterogeneity within countries. National coverage is a births-weighted average of subpopulations with different contact and attrition hazards, and herd protection is a local, not national, property; a national rest point within the herd band is compatible with subpopulations far below it, where outbreaks originate (25, 26). The wealth gradient in full immunization and zero-dose prevalence documented across household surveys (27–29), and the association between subnational gender inequality and immunization coverage (30, 31), reflect this heterogeneity. The second theme is gender. In most settings, the vaccination decision is made by mothers, whose time cost and mobility determine both the full cost of vaccinating and the probability of routine contact with the health system (32, 33); gender therefore enters the model through the cost term and the contact hazard, and the study draws out what the aggregate estimates can and cannot say about this channel.

The study contributes to three literatures. In health economics, it offers, to my knowledge, the first direct estimation of the vaccination externality's dynamic law on the global coverage panel, together with a decomposition of that law into a payoff-responsive and a payoff-insensitive component with sharply different policy levers. To economic epidemiology (34), it converts the behavior-epidemiology feedback literature (35, 36) from a simulation exercise into an estimation exercise with explicit identification. To apply evolutionary game theory, it demonstrates that the replicator-mutator equation is estimable using official statistics, that its boundary structure, usually treated as a nuisance, is precisely what identifies the institutional flows, and that its policy content survives the change of revision protocol that separates imitation dynamics from best-response dynamics (16, 37). The remainder of the study is organized as follows. Section 2 describes the data, derives the model from household choice through aggregation to the estimating equation, and states the identification and timing results. Section 3 reports the results, including the leverage arithmetic implied by the estimates. Section 4 discusses interpretation, alternative readings of the linear force, health systems, policy, gender, and limitations. Section 5 concludes.

## 2 Materials and methods

### 2.1 Data

The core dataset is the WHO/UNICEF Estimates of National Immunization Coverage, 2025 revision, released in July 2026, which reports the estimated share of each national birth cohort (or surviving-infant cohort) receiving each antigen in each calendar year (10). I use six antigens: the first and third doses of the diphtheria-tetanus-pertussis series (DTP1, DTP3), the first and second doses of measles-containing vaccine (MCV1, MCV2), the third dose of hepatitis B (HepB3), and the third dose of *Haemophilus influenzae* type b (Hib3). The two DTP margins separate first contact with the immunization system from completion of a multi-dose schedule; MCV1 is the analysis's headline antigen because measles has the highest basic reproduction number among the vaccine-preventable diseases and therefore the most demanding herd threshold; HepB3 and Hib3 are staggered-introduction antigens whose national rollouts traverse the entire coverage range within the sample window. Polio antigens are deliberately excluded: the 2025 WUENIC revision redefined the polio series, and polio immunity in much of the sample period was driven by eradication campaigns rather than routine choice, which is the margin the model describes. Coverage is divided by 100 and capped at 0.995 to keep the share strictly inside the unit interval. The estimation panel for MCV1 comprises 8,019 country-year first differences across all 195 countries.

Three auxiliary sources complete the dataset. The WUENIC production input file provides, for each country-antigen-year, administrative coverage reported by national programs, the government's official estimate, the working group's grade of confidence, and the UN cohort denominators. I use the administrative series and confidence grades to assess robustness. The UN World Population Prospects 2024 provides annual births and total population, which serve as cohort weights. The World Bank's World Development Indicators provide purchasing-power-parity GDP per capita and current health expenditure per capita as income and health system capacity controls; the health expenditure series begins in 2000. Finally, the WHO Joint Reporting Form's global reported measles case series motivates the welfare stakes descriptively; because the extract available to this study is the global aggregate rather than the country panel, reported cases enter the analysis only descriptively, and the estimation of outbreak-responsiveness at the country level is left to future work with country-level surveillance data. The panel is national: it carries no disaggregation by wealth, residence, caste, ethnicity, or the sex of the caregiver, so within-country heterogeneity is treated theoretically in Sections 2.3 and 4.5 and empirically only through the cross-country income gradient.

### 2.2 A microeconomic model of vaccination choice

A household decides whether the child completes a vaccination schedule (*V*) or not (*N*). Health enters utility as a capital stock in the sense of Grossman (38); infection depreciates the stock. Let *L* > 0 denote the expected utility loss from infection, and let the annual force of infection faced by an unvaccinated child be


$$\begin{array}{l} \lambda \left(x\right)=\lambda_{0}\left(1-\theta x\right),\;\theta \in \left(0,1\right), \end{array}$$ (1)

which declines in the vaccinated share *x* of the community: this is herd protection, the mass-action externality of the epidemiological workhorse models (39, 40). Vaccination reduces the household's exposure to a fraction (1 − *e*) of λ(*x*), where *e* is effectiveness, at a total cost *c* that bundles the money price, the time and travel price, and the psychological cost that the hesitancy literature documents as heterogeneous and shock-sensitive (11, 12). The expected payoff difference between vaccinating and not vaccinating is therefore


$$\begin{array}{l} \Delta \pi \left(x\right)=\pi_{V}\left(x\right)-\pi_{N}\left(x\right)=eL\lambda_{0}\left(1-\theta x\right)-c, \end{array}$$ (2)

which is strictly decreasing in *x* whenever θ > 0. Equation 2 contains the two comparative statics that organize the empirical work. First, the free-riding signature: the private return to vaccination falls as the community's coverage rises, so any payoff-responsive adjustment process decelerates endogenously. Second, the Nash-vs.-planner gap: an atomistic household sets Δπ(*x*) = 0, giving the interior Nash coverage


$$\begin{array}{l} x^{N}=\frac{1}{\theta }\left(1-\frac{c}{eL\lambda_{0}}\right), \end{array}$$ (3)

whereas a planner who internalizes the infections each vaccination prevents values the marginal vaccination at Δπ(*x*) plus a strictly positive external term and therefore demands coverage up to the herd threshold $\bar{x}=1-1/R_{0}$ whenever elimination is feasible at cost *c* (1, 2, 4). For measles, with *R*_0_ between 12 and 18, the threshold band is 91.7%–94.4% (21); throughout the study I use 92%–94% as the herd band. The prediction to test is not the level of coverage but the shape of its law of motion: a payoff-responsive population should exhibit negative frequency dependence, and its flow should vanish at an interior rest point below the band.

Two features of the cost term matter for what follows. First, households are heterogeneous: the cost of vaccinating a household *i* is *c*_*i*_ = *p* + *w*_*i*_τ_*i*_ + ψ_*i*_, where *p* is the money price, τ_*i*_ the time a completed schedule requires of the caregiver, *w*_*i*_ is the shadow value of that time, and ψ_*i*_ is a psychological or informational cost; *c*_*i*_ is drawn from a distribution *F* with mean $\bar{c}$. Because Δπ_*i*_(*x*) = *eLλ*_0_(1 − θ*x*) − *c*_*i*_ is affine in *c*_*i*_, the population-average payoff difference is $\Delta \bar{\pi }\left(x\right)=eL\lambda_{0}\left(1-\theta x\right)-\bar{c}$, and heterogeneity leaves the affine form of Equation 2 intact while shifting its level; this is what allows the aggregate law of motion in Section 2.3 to inherit the structure of the household problem. Second, the decision is typically executed by the mother, who bears τ_*i*_ and whose mobility, autonomy, and time poverty determine both *w*_*i*_τ_*i*_ and whether the household is in contact with the health system at all (32, 33, 41, 42). In a collective household in which the decision-maker's valuation *L*_*d*_ and the caregiver's time cost enter with bargaining weights, the effective cost rises with the caregiver's time price and the effective benefit falls when the decision rests with a member who values child health less or discounts the caregiver's information; gender norms therefore enter the model through *c*_*i*_ and, as Section 2.3 makes explicit, through the contact probability that governs the enrollment hazard. The national panel does not identify these parameters separately; their role is to make precise which channel the gender evidence discussed in Section 4.5 speaks to.

### 2.3 From choice to population dynamics: aggregation and the health system

Households do not re-optimize annually, and the aggregate law of motion comes from an explicit revision process rather than being assumed. Consider a large population of households indexed by *i*, each holding one of the two states *V* or *N*, with costs *c*_*i*_ drawn from *F*. Three events can change a household's state in a given year, and their probabilities define the model's three flows.

The first event is payoff-responsive revision. With probability σ a household receives a revision opportunity, observes the choice and outcome of another household drawn at random, and, if the two choices differ, switches to the other's choice with a probability that is affine in the payoff difference between the two choices as evaluated by the revising household. This is the pairwise proportional imitation protocol whose mean dynamic is the replicator equation (16, 43, 44). The expected net flow from *N* to *V* is σ times the probability that a random pair is discordant, *x*(1 − *x*), times the mean payoff difference across such pairs; because Equation 2 is affine in *c*_*i*_, that mean equals $\Delta \bar{\pi }\left(x\right)=eL\lambda_{0}\left(1-\theta x\right)-\bar{c}$ up to a truncation term that vanishes when the sign of Δπ_*i*_(*x*) is common across households in the relevant window. The selection term therefore inherits the affine payoff structure of the household problem, evaluated at the mean cost, and the variance factor *x*(1 − *x*) is not an assumption but the probability that a revising household encounters a household that made the other choice.

The second event is institutional enrollment. Independently of any payoff comparison, an unvaccinated-track child comes into contact with the health system with annual probability ρ (birth registration, well-child visits, outreach sessions, school entry) and, conditional on contact, is enrolled in the schedule with probability *q*. The enrollment hazard is μ_*in*_ = ρ*q*. The third event is attrition: a vaccinated-track household leaves the track with annual probability μ_*out*_ = *m* + *d* + *k*, where *m* collects migration and demographic churn, *d* provider discontinuity, stock-outs and administrative failure, and *k* confidence and misinformation shocks. Letting the population grow large, the stochastic process converges over any finite horizon to its mean dynamic (16, 45), which is the two-strategy replicator-mutator equation (13–15, 46):


$$\begin{array}{l} ẋ=\underset{\text{selection}}{\underset{︸}{\sigma x\left(1-x\right)\Delta \bar{\pi }\left(x\right)}}+\underset{\text{mutation}}{\underset{︸}{\rho q\left(1-x\right)-\mu_{out}x}}. \end{array}$$ (4)

Equation 4 locates the health system in the model. The payoff channel carries the household's economics: perceived severity *L*, effectiveness *e*, the money, time, and psychological components of $\bar{c}$, and the revision rate σ that measures how salient the decision is. The mutation channel carries the system's institutions: the contact probability ρ depends on workforce density, facility reach and outreach, the conversion probability *q* on vaccine availability, default scheduling and provider practice, and the attrition components *d* and *k* on continuity of care, information systems and public trust. Table 1 maps each parameter to the health-system building block that governs it and to the class of policy instrument that moves it (17, 47). The mapping makes the article's main empirical question sharp: if governance, financing, and workforce are the leading determinants of service coverage, as the recent panel literature finds (18–20), they should act on coverage dynamics through ρ, *q* and *d*, that is, through the linear terms of Equation 4, and not through the variance-weighted payoff term. Section 3 shows where the data place them.

**Table 1:** Model parameters, their health-system determinants and the instruments that move them.

Parameter	Meaning in the model	Governing health-system component	Instrument class	Enters the law of motion through
λ_0_, θ	Force of infection and strength of herd protection	Epidemiology (*R*_0_), transmission setting	None directly	Payoff term, slope
*e*	Vaccine effectiveness as experienced	Medical products, cold chain, schedule adherence	Supply quality	Payoff term, level
*L*	Perceived utility loss from infection	Information, surveillance, risk communication	Payoff instrument	Payoff term, level
*p*	Money price to the household	Financing (user fees, insurance)	Subsidy, fee removal, conditional transfer	Payoff term, level
*wτ*	Time and travel price borne by the caregiver	Service delivery (distance, waiting, session timing), caregiver time poverty	Outreach, scheduling, transport	Payoff term, level; also ρ
ψ	Psychological and informational cost	Trust, misinformation environment	Communication, community engagement	Payoff term, level
σ	Revision rate: salience of the decision	Information systems, campaigns	Campaigns	Scale of the payoff term
ρ	Annual probability of contact with the system	Workforce density, facility reach, outreach, birth-registration linkage	Contact instrument	Mutation term, enrollment hazard μ_in_ = ρ*q*
*q*	Probability that contact converts to enrollment	Medical products (stock), default scheduling, provider practice, governance	Conversion instrument	Mutation term, enrollment hazard μ_in_ = ρ*q*
*m*	Migration and demographic churn	Population registers, mobile-population services	Continuity instrument	Mutation term, attrition hazard μ_out_ = *m* + *d* + *k*
*d*	Provider discontinuity, stock-outs, administrative failure	Financing, medical products, governance	Continuity and supply instrument	Mutation term, attrition hazard μ_out_ = *m* + *d* + *k*
*k*	Confidence and misinformation shocks	Governance, trust, information systems (reminder and recall)	Retention instrument	Mutation term, attrition hazard μ_out_ = *m* + *d* + *k*

Sources: model of Sections 2.2 and 2.3; health-system building blocks after (17); determinant categories after (47) and (20).

### 2.4 Properties of the law of motion

Three properties of Equation 4 drive everything that follows, and a fourth establishes that the policy content does not depend on the imitation protocol.

#### 2.4.1 Proposition 1 (interior attractor)

With $\Delta \bar{\pi }$ affine and decreasing as in Equation 2 and μ_*in*_, μ_*out*_ > 0, the right-hand side of Equation 4 is positive at *x* = 0, negative at *x* = 1, and its relevant branch crosses zero once from above on (0, 1); hence there exists a stable interior rest point x^*^, and coverage converges to it at the local rate |*f*′(x^*^)|, where *f* denotes the right-hand side of Equation 4. The proof is by sign inspection of the cubic *f* and is standard (48).

#### 2.4.2 Proposition 2 (mutation-supported ceiling)

Even if $\Delta \bar{\pi }\left(x\right)>0$ for all *x*, so that selection alone would carry coverage to one, the rest point satisfies $x^{*}\leq \mu_{in}/\left(\mu_{in}+\mu_{out}\right)+\sigma \Delta \bar{\pi }x^{*}\left(1-x^{*}\right)/\left(\mu_{in}+\mu_{out}\right)<1$ whenever μ_*out*_ > 0. Attrition places a ceiling on coverage that no payoff improvement can fully remove, because the payoff channel is throttled by the vanishing factor *x*(1 − *x*) near the boundary. This is the population-level analog of the mutation-supported stationary states of Kandori et al. (49).

#### 2.4.3 Proposition 3 (policy leverage)

Consider two marginal instruments at the rest point: a payoff instrument that raises $\sigma \Delta \bar{\pi }$ uniformly by *dΔ* (a subsidy, a conditional transfer, or a risk-information campaign) and a retention instrument that lowers μ_*out*_ by *dμ* (defaults, reminders, or school-entry verification). Implicit differentiation of *f*(x^*^) = 0 gives


$$\begin{array}{r} \frac{dx^{*}}{d\Delta }=\frac{x^{*}\left(1-x^{*}\right)}{\left|f^{\prime }\left(x^{*}\right)\right|},\;\frac{dx^{*}}{d\mu }=\frac{x^{*}}{\left|f^{\prime }\left(x^{*}\right)\right|}, \end{array}$$ (5)

$$\begin{array}{r} \frac{dx^{*}/d\mu }{dx^{*}/d\Delta }=\frac{1}{1-x^{*}}. \end{array}$$

At x^*^ = 0.876, the estimated median rest point, the retention instrument is 8.1 times more powerful per unit than the payoff instrument, and the ratio grows without bound as x^*^ → 1. The last mile of herd immunity is structurally a retention problem.

#### 2.4.4 Remark 1 (protocol dependence of the linear term)

The imitation protocol is not the only revision process consistent with the household model. Under a perturbed best-response protocol, a revising household chooses *V* with probability *P*(Δπ(*x*)), where *P* is a smooth, non-decreasing choice function; the logit case *P*(*u*) = 1/(1 + *e*^ − κ*u*^) is the leading example (37, 50, 51). The mean dynamic with mutation is then $f\left(x\right)=\sigma \left[P\left(\Delta \bar{\pi }\left(x\right)\right)-x\right]+\mu_{in}\left(1-x\right)-\mu_{out}x$. Two consequences follow. First, if *P* is locally flat at $\bar{P}$ over the observed coverage window, $f\left(x\right)=\left(\sigma \bar{P}+\mu_{in}\right)-\left(\sigma +\mu_{in}+\mu_{out}\right)x$, which is observationally equivalent to pure mutation with ${\tilde{\mu }}_{in}=\sigma \bar{P}+\mu_{in}$ and ${\tilde{\mu }}_{out}=\sigma \left(1-\bar{P}\right)+\mu_{out}$: the linear restoring coefficient then measures total turnover, institutional and revisional, and the hazards recovered from it are upper bounds on the institutional flows. Second, if *P* is payoff-responsive, linearizing around x^*^ gives $f^{\prime }\left(x^{*}\right)=-\sigma \left[1+P^{\prime }\left(\Delta \bar{\pi }\left(x^{*}\right)\right)eL\lambda_{0}\theta \right]-\left(\mu_{in}+\mu_{out}\right)$, so free riding steepens the restoring force instead of appearing as a separate variance-weighted term. The two protocols differ only in whether the payoff contribution is weighted by *x*(1 − *x*); on a narrow interior window they cannot be told apart, whereas on data that traverse the simplex they can, because under imitation the payoff contribution must vanish at both corners. This is why the rollout antigens, and not the interior, discriminate between the two readings, and why “payoff-blind” is used in this study in the operational sense of payoff-insensitive over the observed window.

#### 2.4.5 Proposition 4 (protocol robustness of the leverage ordering)

Let the revision flow be $\sigma \left[P\left(\Delta \bar{\pi }\left(x\right)\right)-x\right]$ for any differentiable, non-decreasing choice function *P* bounded in [0, 1] with *P*′ → 0 as *P* → 1, which holds for the logit, the probit, and any protocol derived from an unbounded taste-shock distribution, and add the mutation flows of Equation 4. At an interior rest point, $dx^{*}/d\Delta =\sigma P^{\prime }\left(\Delta \bar{\pi }\left(x^{*}\right)\right)/|f^{\prime }\left(x^{*}\right)|$ and dx^*^/*dμ* = x^*^/|*f*′(x^*^)|, so the leverage ratio is x^*^/(σ*P*′). Under the logit protocol *P*′ = κ*P*(1 − *P*), and since $P\left(\Delta \bar{\pi }\left(x^{*}\right)\right)$ approaches x^*^ as the mutation hazards become small, the ratio is approximately 1/[σκ(1 − x^*^)]: the shape of Equation 5 is preserved, and only its scale is set by responsiveness. More generally, along any path on which x^*^ → 1 the derivative *P*′ vanishes while dx^*^/*dμ* converges to 1/|*f*′(1)| > 0, so the leverage of retention over payoff instruments diverges for every protocol in the class. The variance throttle of the replicator equation is thus a special case of a general fact: any bounded choice probability must flatten near a corner, and a payoff shift acts on coverage only through that slope.

### 2.5 Estimating equation and structural mapping

Discretizing Equation 4 at the annual frequency and allowing the payoff level to vary by country and year yields the estimating equation:


$$\begin{array}{l} \Delta x_{it}=\beta_{0}s_{i,t-1}+\beta_{1}s_{i,t-1}x_{i,t-1}+\gamma_{1}x_{i,t-1}+\alpha_{i}+\tau_{t}+\varepsilon_{it}, \end{array}$$ (6)

$$\begin{array}{l} s_{i,t-1}\equiv x_{i,t-1}\left(1-x_{i,t-1}\right), \end{array}$$

where Δ*x*_*it*_ = *x*_*it*_ − *x*_*i,t*−1_. The mapping to structure is exact: the selection channel contributes $\beta_{0}=\sigma \left(eL\lambda_{0}-\bar{c}\right)$ and β_1_ = −σ*eLλ*_0_θ, so that β_1_ < 0 is the free-riding signature, and the ratio $\beta_{0}/\left(-\beta_{1}\right)=x^{N}$ recovers the Nash coverage of Equation 3, the level at which selection alone would come to rest; the mutation channel contributes the linear terms, with α_*i*_ absorbing the country-specific enrollment hazard μ_*in,i*_ = ρ_*i*_*q*_*i*_ and γ_1_ = −(μ_*in*_ + μ_*out*_), so that the linear restoring coefficient measures total behavioral turnover, the annual probability mass that crosses between the two states for payoff-insensitive reasons. Standard errors are clustered by country throughout; weighted specifications use annual births.

### 2.6 Identification

The claim that boundary observations identify the mutation hazards separately from selection can be stated exactly. Writing the deterministic part of Equation 6 for a given country as $f\left(x\right)=\alpha +\gamma_{1}x+\beta_{0}x\left(1-x\right)+\beta_{1}x^{2}\left(1-x\right)$ and expanding in powers of *x*,


$$\begin{array}{l} f\left(x\right)=a_{0}+a_{1}x+a_{2}x^{2}+a_{3}x^{3}, \end{array}$$ (7)

$$\begin{array}{l} a_{0}=\alpha ,a_{1}=\gamma_{1}+\beta_{0},a_{2}=\beta_{1}-\beta_{0},a_{3}=-\beta_{1}. \end{array}$$

#### 2.6.1 Proposition 5 (identification by boundary values)

The map from (α, γ_1_, β_0_, β_1_) to the cubic coefficients (*a*_0_, *a*_1_, *a*_2_, *a*_3_) is linear and invertible, with inverse α = *a*_0_ = *f*(0), γ_1_ = *a*_1_ + *a*_2_ + *a*_3_ = *f*(1) − *f*(0), β_1_ = −*a*_3_ and β_0_ = −(*a*_2_ + *a*_3_). Under the imitation protocol, the enrollment hazard is the value of the law of motion at zero coverage, and the turnover coefficient is the difference between its values at full and at zero coverage, two quantities that contain no payoff parameter because the selection term vanishes at both corners; the payoff parameters are, conversely, identified only from the curvature of *f*. The reduced-form regression (Equation 6) is therefore the structural equation in a different basis, and no separate structural estimation can add identifying content: the deep parameters $\left(\sigma ,e,L,\lambda_{0},\theta ,\bar{c}\right)$ enter only through the two composites β_0_ and β_1_, which are exactly the objects that Propositions 1 to 3 require.

Four consequences organize the empirical work. First, the precision of γ_1_ depends on whether the corner values *f*(0) and *f*(1) are interpolated or extrapolated. For MCV1, they are interpolated: coverage in the early 1980s started near zero in many countries and reached the 0.99 cap in others, so the binned means reported in Section 3.2 span the whole simplex, and the turnover coefficient is estimated with a *t*-statistic above 10. Second, the payoff slope is a curvature parameter, and curvature is poorly determined when 83% of the observations sit between 0.60 and 0.99; the correlation of 0.873 between *s* and *sx* in the MCV1 sample is the symptom of this, not a separate problem to be solved by orthogonalization. What the interior does determine well is the local payoff level $\beta_{0}+\beta_{1}\bar{x}$ at the center of the window, which is the tangent of the payoff term, whereas the slope β_1_ requires support at both ends of the simplex. The rollout antigens supply that support: the staggered national introductions of Hib and HepB vaccines were timed by Gavi financing windows and WHO recommendations rather than by household payoffs, so they place countries at every point of the simplex at dates that are plausibly orthogonal to household demand shocks, and it is this externally timed variation in support, not an instrument for the regressor, that identifies β_1_. Third, the identification is parametric. It rests on the affine payoff function (Equation 2), which makes *f* a cubic; a payoff function with curvature would add higher-order terms, and the binned means reported in Section 3.2 are visually consistent with a cubic but do not test it, so the result should be read as identification under the model's functional form. Fourth, as Remark 1 makes explicit, the corner values identify institutional hazards under the imitation protocol; under a best-response protocol they identify total inflow and outflow at the corners, revision included, and the recovered hazards are upper bounds.

Instrumental variables are not the natural remedy for the remaining threats to Equation 6, and it is worth saying why. The regressors are deterministic functions of the lagged state, so Equation 6 is a non-linear autoregression: the state does not become endogenous through simultaneity, and any external variable that shifts coverage does so through α_*i*_ or through $\Delta \bar{\pi }$, that is, through the equation itself, which is incompatible with an exclusion restriction. The two genuine threats are measurement error in the lagged share, which generates division bias under contemporaneous dating and attenuation under lagged dating, and the dynamic-panel bias of Nickell (52). The first is addressed by dating the regressors at *t* − 2, which is the internal-instrument logic of the errors-in-variables panel literature (53, 54): under serially uncorrelated measurement error, the twice-lagged share is a valid instrument for the once-lagged share, and I report the twice-lagged estimate as a conservative bound. The second is bounded by the time dimension and checked against the pooled estimator, which is free of it. Section 2.7 states the resulting tests.

### 2.7 Timing and measurement

Two econometric hazards require explicit handling. The first is division bias by timing. Because Δ*x*_*it*_ = *x*_*it*_ − *x*_*i,t*−1_, regressing the change on functions of the contemporaneous share mechanically loads the dependent variable's own innovation onto the regressor and reverses the sign of the linear term; the lagged dating in Equation 6 removes the mechanical channel, and a twice-lagged dating removes, to first order, the residual contamination from serially correlated measurement error in the share itself. I report all three datings; the ordering of the estimates is itself a specification test, and the twice-lagged estimate serves as a conservative lower bound on turnover. The second hazard is the dynamic-panel bias of Nickell (52), which biases γ_1_ downward under fixed effects; with an average time dimension above 40 years, the bias is an order of magnitude smaller than the estimates, and the pooled specification without fixed effects, which is free of it, still yields a strongly negative γ_1_. Finally, because WUENIC is itself a smoothed, rule-based reconciliation of reported and survey data (10), part of any measured year-to-year movement is measurement rather than behavior; I bound this by re-estimating Equation 6 on the unsmoothed administrative coverage series and on the subsample restricted to estimates graded as adequately supported by the WUENIC working group.

## 3 Results

### 3.1 Descriptive statistics and the aggregate stylized fact

Table 2 summarizes the six antigen panels. Coverage means range from 79% (MCV1) to 89% (DTP1), with standard deviations near 0.2; mean annual changes are positive but small, between 0.6 and 1.2 points, against within-panel standard deviations of annual change of 5 to 9 points, so the panel is dominated by fluctuation around slowly moving levels rather than by trend. Figure 1 shows the aggregate stylized fact that motivates the welfare question. Births-weighted world MCV1 coverage rose steeply through the 1980s, drifted within a narrow corridor of 70%–86% for three decades, fell visibly after 2019, and at no point approached the 92%–94% herd band; reported global measles cases, meanwhile, never fell to zero and have resurged since 2022. World coverage does not look like a variable in transit toward a target; it looks like a variable orbiting a rest point below the target. The remainder of the section estimates the rest point and the forces that maintain it.

**Table 2:** Descriptive statistics by antigen panel.

Antigen	Obs. (Δx)	Countries	Years	Mean x	SD x	Mean Δx	SD Δx
DTP1	8,145	195	1981–2025	0.885	0.152	0.006	0.051
DTP3	8,145	195	1981–2025	0.804	0.216	0.009	0.068
MCV1	8,019	195	1981–2025	0.793	0.213	0.010	0.076
MCV2	3,444	191	2001–2025	0.820	0.192	0.008	0.081
HepB3	4,753	191	1990–2025	0.848	0.177	0.012	0.086
Hib3	3,983	194	1992–2025	0.863	0.159	0.010	0.079

Coverage x is the WUENIC estimate divided by 100 and capped at 0.995. Sources: WUENIC 2025 revision; author's calculations.

**Figure 1:**
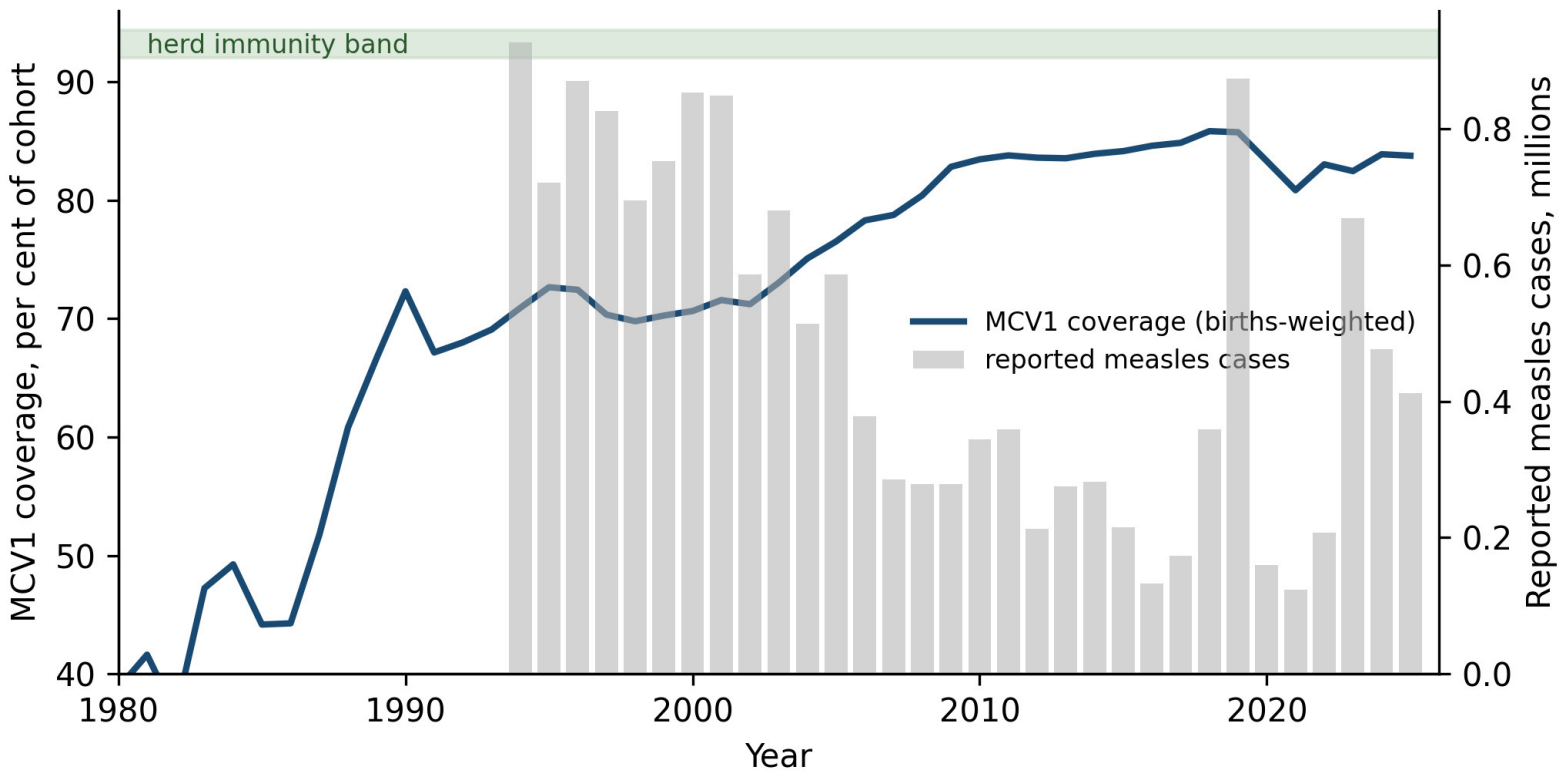
Estimated laws of motion for the median country before 2020 (solid) and from 2020 onward (dashed). Open circles mark the stable rest points, 0.891 and 0.858; the shaded band is the herd immunity band.

### 3.2 The turnover channel dominates the interior

Table 3 reports Equation 6 for MCV1 across five specifications: pooled, country fixed effects, two-way fixed effects, two-way fixed effects with the income and capacity interactions, and the births-weighted two-way specification. The result that survives every specification is the linear restoring term. Its coefficient is −0.110 (*t* = −6.9) in the pooled specification, −0.156 (*t* = −9.3) with country effects, −0.248 (*t* = −10.4) with two-way effects, and −0.172 (*t* = −6.0) births-weighted. Under the structural mapping, this is −(μ_*in*_ + μ_*out*_): between 15 and 25% of the relevant behavioral pool crossing between the vaccinated and unvaccinated tracks each year for payoff-insensitive reasons. For the median country, the two-way estimates decompose the flow into an enrollment hazard ${\hat{\mu }}_{in}=0.203$, the annual probability that an unvaccinated-track child is pulled into the schedule by institutional contact, and an attrition hazard ${\hat{\mu }}_{out}=0.045$, the annual probability that a vaccinated-track household drops out. In the notation of Proposition 5, these are the corner values of the estimated cubic, $\hat{f}\left(0\right)=0.203$ and $\hat{f}\left(1\right)=-0.045$, and the turnover coefficient is their difference; the remaining cubic coefficients for the median country are *a*_1_ = −0.137, *a*_2_ = −0.088 and *a*_3_ = −0.023, so the two curvature terms that carry the payoff parameters are smaller than the restoring coefficient of −0.248, the cubic coefficient by an order of magnitude, and the quadratic coefficient by a factor of about three. As Remark 1 cautions, the hazards are upper bounds on the institutional component if part of the interior turnover is payoff-insensitive revision. Coverage is high not because households continuously find vaccination optimal but because the institutional default recruits a fifth of the uncovered pool every year, four and a half times faster than drift erodes the covered pool.

**Table 3:** The law of motion of MCV1 coverage, 1981–2025.

Dependent variable: Δx	(S1) Pooled	(S2) Country FE	(S3) Two-way FE	(S4) + controls	(S5) Weighted
Selection level, s (lagged)	0.031 (0.39)	0.183 (2.13)	0.111 (1.21)	−1.116 (−1.40)	0.097 (0.70)
Frequency dependence, s·x (lagged)	−0.063 (−0.64)	−0.080 (−0.74)	0.023 (0.20)	1.095 (1.53)	−0.040 (−0.26)
Linear restoring term, x (lagged)	−0.110 (−6.90)	−0.156 (−9.25)	−0.248 (−10.38)	−0.578 (−2.72)	−0.172 (−6.02)
Income interaction, s × ln GDPpc				−0.192 (−3.02)	
Capacity interaction, s × ln CHEXpc				0.114 (2.77)	
Country fixed effects	No	Yes	Yes	Yes	Yes
Year fixed effects	No	No	Yes	Yes	Yes
Birth weights	No	No	No	No	Yes
Observations	8,019	8,019	8,019	4,428	8,019

*t*-statistics in parentheses, standard errors clustered by country (195 clusters). The income and capacity interactions demean log GDP per capita (PPP) and log current health expenditure per capita (current US$). Column S4 loses observations due to control availability, since the health expenditure series begins in 2000.

The selection terms tell the opposite story. The level term β_0_ is positive and significant only before year effects enter (0.183, *t* = 2.1, column S2) and loses significance under two-way effects (0.111, *t* = 1.2); the frequency-dependence term β_1_ is never significant in the interior and even changes sign across columns, negative in S1, S2 and S5 and positive in S3 and S4, without approaching significance, exactly the behavior that Section 2.6 predicts of a curvature coefficient estimated on a narrow window. The combination that the interior determines, the local payoff level $\beta_{0}+\beta_{1}x^{*}$ at the median rest point, is 0.131 in flow units under two-way effects, positive but modest relative to the enrollment hazard of 0.203. It is also positive in the fixed-effects columns without controls (0.113 in S2, 0.062 in the births-weighted S5) but negative (−0.157) in column S4, which is estimated on the post-2000 subsample with the income and capacity interactions, so even this combination is held loosely by the interior. The payoff shifters in column S4 are nonetheless informative: conditional on income, health system capacity raises the drift toward vaccination significantly (the capacity interaction is 0.114, *t* = 2.77), while income conditional on capacity enters negatively (−0.192, *t* = −3.02), so the cross-sectional association usually attributed to income operates through the health system rather than through household resources, which is the pattern Table 1 predicts if the leading determinants of coverage act through the contact and conversion probabilities rather than through willingness to pay. The honest reading is not that payoffs do not matter but that on the narrow interior window the panel cannot separate the level of the payoff difference from its slope, while it measures the linear turnover force with great precision. Figure 2 makes the point visually: the binned annual change declines monotonically in lagged coverage, crosses zero at the median-country rest point of 0.88, and is tracked closely by the fitted model, and the crossing occurs below the herd band.

**Figure 2:**
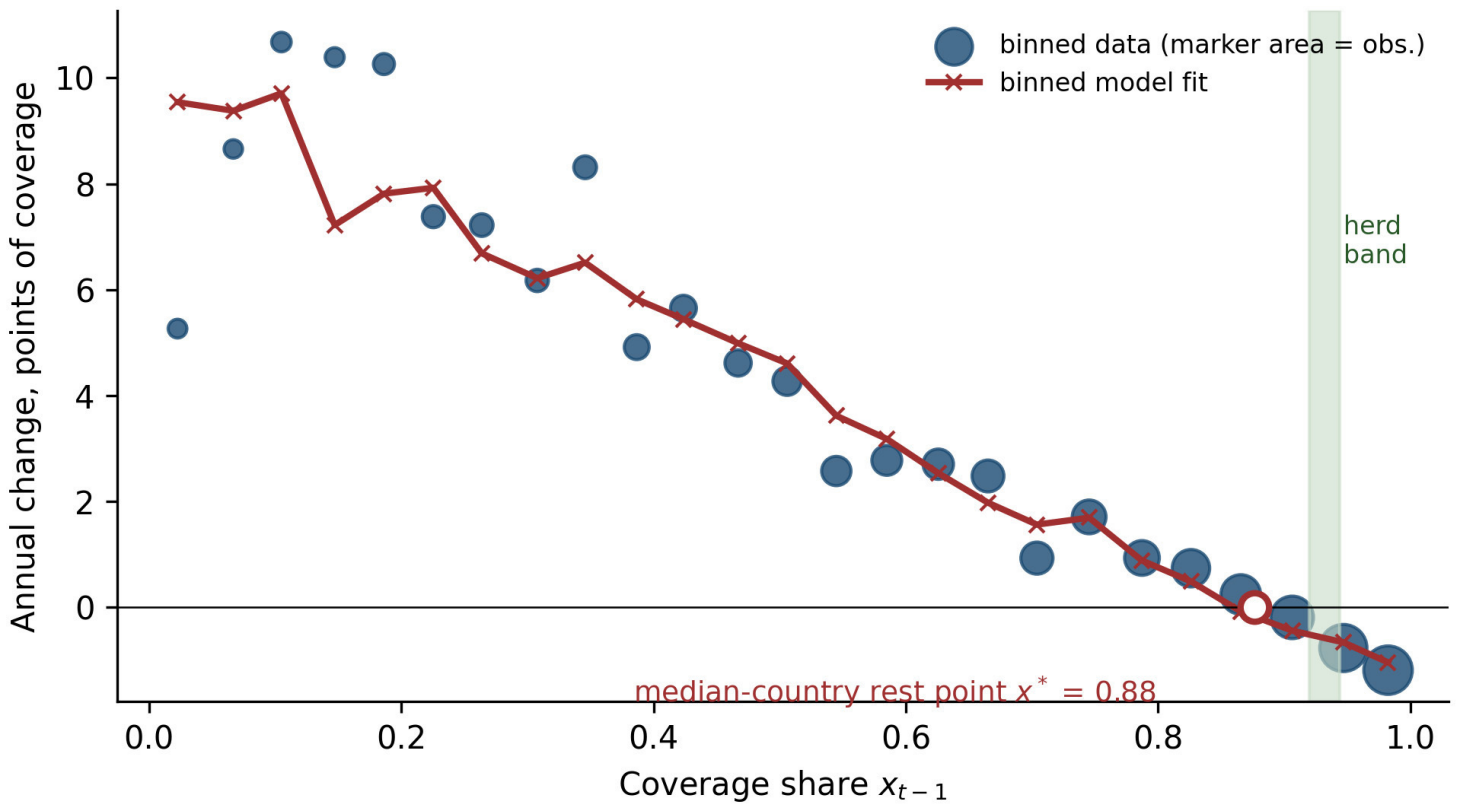
Distribution of estimated country rest points for MCV1 against the herd immunity band (shaded). The dashed line marks the births-weighted world mean rest point of 0.78.

### 3.3 Where selection is identified: the rollout antigens

Table 4 estimates the two-way specification antigen by antigen, and the cross-antigen pattern is the article's second main result. The completion margins, DTP3, MCV1, MCV2, HepB3, and Hib3, all display the strong linear restoring force, with coefficients from −0.168 (DTP3) to −0.475 (MCV2), each with a *t* statistic of at least 3.6. The single exception is DTP1, the first-contact margin, whose linear term is a precisely estimated zero (−0.039, *t* = −0.85): once a household has ever been reached, there is no payoff-insensitive force pulling the first dose away, which is exactly what the model predicts of a margin with no completion component to erode. Instead, DTP1 is the margin where selection is visible: the level term is 0.588 (*t* = 2.98) and the frequency-dependence term is negative and marginally significant (−0.346, *t* = −1.80). The implied Nash coverage β_0_/(−β_1_) for DTP1 exceeds one, so the private return to first contact remains positive over the whole coverage range: selection alone would not stall the first dose, and the DTP1 shortfall that persists in many countries is therefore a contact problem, a low ρ, rather than a free-riding problem.

**Table 4:** The law of motion by antigen, two-way fixed effects.

Dependent variable: Δx	DTP1	DTP3	MCV1	MCV2	HepB3	Hib3
Selection level, s (lagged)	0.588 (2.98)	0.218 (2.80)	0.111 (1.21)	−0.332 (−1.64)	0.257 (0.84)	0.833 (2.58)
Frequency dependence, s·x (lagged)	−0.346 (−1.80)	−0.013 (−0.12)	0.023 (0.20)	0.249 (1.08)	−0.174 (−0.52)	−0.959 (−2.97)
Linear restoring term, x (lagged)	−0.039 (−0.85)	−0.168 (−9.21)	−0.248 (−10.38)	−0.475 (−10.26)	−0.339 (−5.24)	−0.296 (−3.60)
Observations	8,145	8,145	8,019	3,444	4,753	3,983

All specifications include country and year fixed effects; *t*-statistics in parentheses, standard errors clustered by country.

Hib3 is the cleanest laboratory because its national rollouts begin at zero within the sample and traverse the entire simplex, giving the selection regressors the support they lack in the interior. There the free-riding signature emerges sharply: β_0_ = 0.833 (*t* = 2.58) and β_1_ = −0.959 (*t* = −2.97). The implied payoff difference declines in coverage and reaches zero at $x^{N}=\beta_{0}/\left(-\beta_{1}\right)=0.87$: selection alone, unassisted by the institutional default, which would have stalled the Hib rollout at 87%, the Nash coverage of Equation 3, below the coverage most programs eventually attained. HepB3, the other staggered-introduction antigen, carries the predicted signs on both selection terms, but neither is significant (0.257, *t* = 0.84; −0.174, *t* = −0.52), so the rollout evidence for the free-riding signature rests on Hib3 alone; comparing the within-sample support of the two rollouts is a robustness exercise left for the extensions. The Hib3 estimates also discriminate between the two revision protocols of Remark 1: the payoff contribution is significant in its variance-weighted form, which is the imitation protocol's prediction and which a flat-choice best-response protocol would not generate. The theory's central mechanism, a private return to vaccination that erodes as protection becomes collective, is therefore not rejected by the data; it is confirmed precisely where the data are capable of testing it, and it is quantitatively strong there.

### 3.4 Timing, division bias, and measurement

Table 5 and Figure 3 subject the turnover estimate to the dating and measurement tests announced in Section 2.7. Dating the right-hand side contemporaneously reverses the sign of the linear term to +0.244 (*t* = +10.3): because the dependent variable is *x*_*t*_ − *x*_*t*−1_, the innovation in *x*_*t*_ sits on both sides and manufactures a positive coefficient mechanically. The reversal is not a robustness failure; it is the fingerprint of division bias, and its magnitude, almost exactly the mirror image of the lagged estimate, indicates how completely the mechanical channel can mask the behavioral one when timing is careless. Dating at two lags, which removes first-order contamination from serially correlated measurement error in the share and plays the role of an internal instrument, yields −0.146 (*t* = −8.8). The behavioral turnover therefore lies between 0.15 and 0.25 per year, with the true value likely nearer the lower bound.

**Table 5:** Timing and measurement of the linear restoring term (MCV1).

Dependent variable: Δx	Contemporaneous (t)	Baseline (t−1)	Two lags (t−2)	Administrative series	Grade-restricted
Selection level, s	0.162 (2.04)	0.111 (1.21)	0.045 (0.58)	−0.637 (−3.37)	−1.617 (−1.59)
Frequency dependence, s·x	−0.245 (−2.48)	0.023 (0.20)	−0.052 (−0.58)	0.648 (3.67)	1.595 (1.77)
Linear restoring term, x	0.244 (10.33)	−0.248 (−10.38)	−0.146 (−8.78)	−0.546 (−9.19)	−0.686 (−2.55)
Observations	8,019	8,019	7,824	4,384	2,895

Two-way fixed effects throughout; t-statistics in parentheses, standard errors clustered by country. The contemporaneous column dates all right-hand variables at t and illustrates division bias: the restoring term's sign reverses. The administrative column replaces WUENIC with national administrative coverage; the grade-restricted column keeps WUENIC country-years with confidence grade of at least 2.

**Figure 3:**
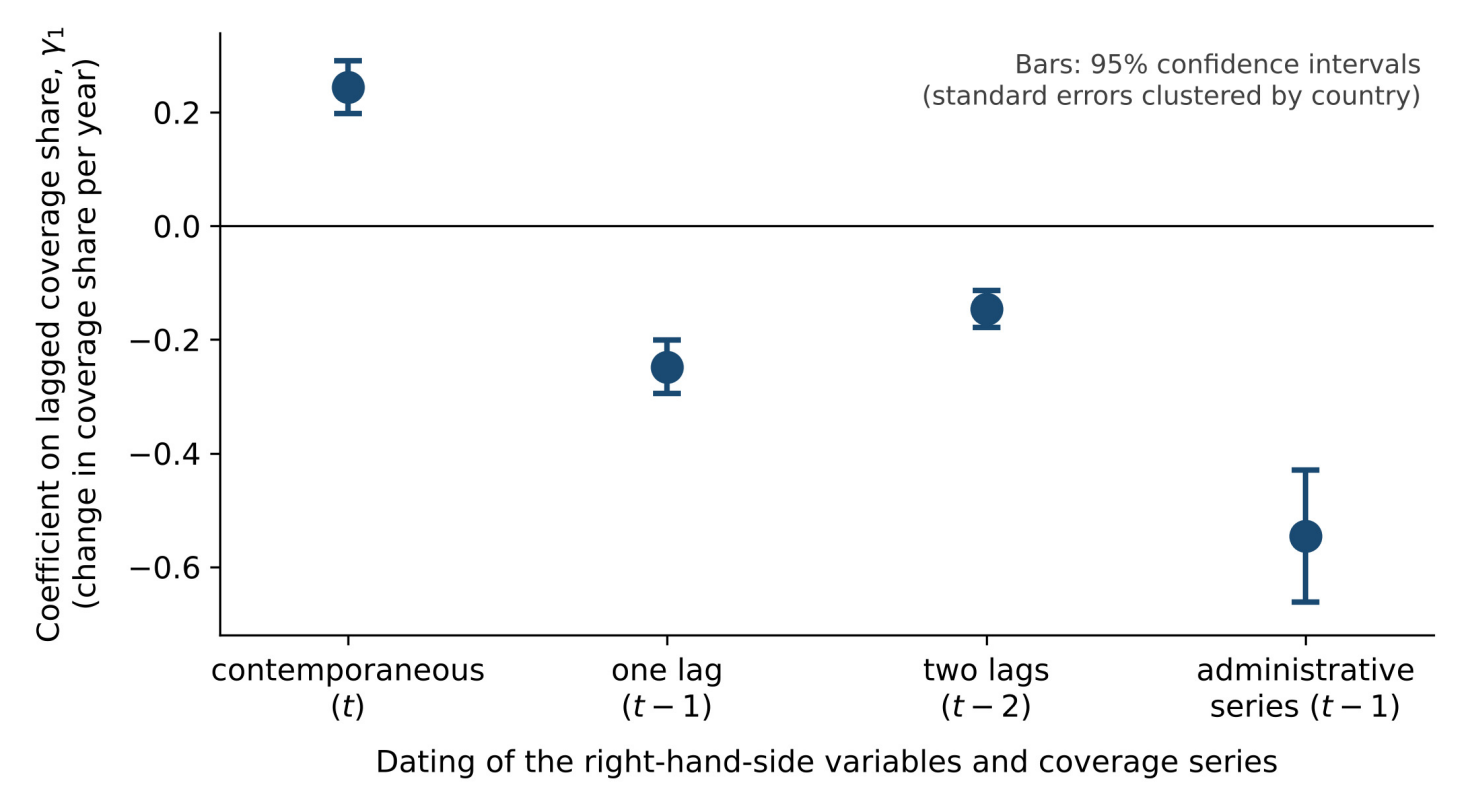
Births-weighted world MCV1 coverage (line, left axis) against the herd immunity band implied by a measles basic reproduction number of 12–18 (shaded) and reported global measles cases (bars, right axis). Sources: WUENIC 2025 revision; WHO reported-case series; UN WPP 2024.

The measurement columns bound the same object from the data-production side. Re-estimating the law of motion on the unsmoothed administrative coverage series roughly doubles the apparent turnover to −0.546 (*t* = −9.2): administrative data are noisier, and noise masquerades as movement, which is precisely why the WUENIC reconciliation exists. Restricting the WUENIC sample to country-years graded as adequately supported by reported and survey data yields −0.686, but this subsample is small (2,895 observations), concentrated in survey-anchored lower-income country-years, and its selection terms are unstable, so I read it as confirming the sign and the order of magnitude rather than the point value. Across all dating and series, the linear restoring force never loses significance. The frequency-dependence term is significant in two columns of Table 5, and neither reinstates the interior free-riding signature: in the contemporaneous column it is negative (−0.245, *t* = −2.48) but carries the same mechanical contamination that reverses the restoring term, and in the administrative column it is significant with the wrong sign (0.648, *t* = 3.67), in a series whose noise the same column shows doubling the apparent turnover. On the reconciled series under lagged dating, no specification yields a significant interior frequency-dependence term.

### 3.5 Rest points and the herd immunity gap

Solving each country's estimated law of motion for its zero, using the country fixed effect as the enrollment hazard and the common selection and restoring coefficients of the two-way specification, yields a stable interior rest point for all 195 countries, approached with a median half-life of 2.0 years. Figure 4 plots the distribution against the herd band, and Table 6 summarizes it by income group. The median rest point is 0.876; the births-weighted world rest point, the coverage level toward which the world's actual birth cohorts gravitate, is 0.780. In all, 74% of countries rest below 0.92, and 91% rest below 0.95. The gradient across income groups is steep and monotonic: the median rest point is 0.676 in low-income countries, where every country rests below the band; 0.754 in lower-middle-income countries; 0.878 in upper-middle-income countries; and 0.917 in high-income countries, where 54% of countries still rest below the band. The herd immunity threshold is thus not a level that the world is slowly approaching; it is a level that the estimated dynamical system does not reach from any income stratum's typical rest point, in high-income countries by a whisker and in low-income countries by 24 points of coverage. Because the restoring coefficient is common and the enrollment hazard is country-specific, the income gradient of rest points is, in the model, a gradient of contact and conversion probabilities: low-income countries rest low not because their households value vaccination less at the margin but because a smaller share of their uncovered pool is reached and enrolled each year.

**Figure 4:**
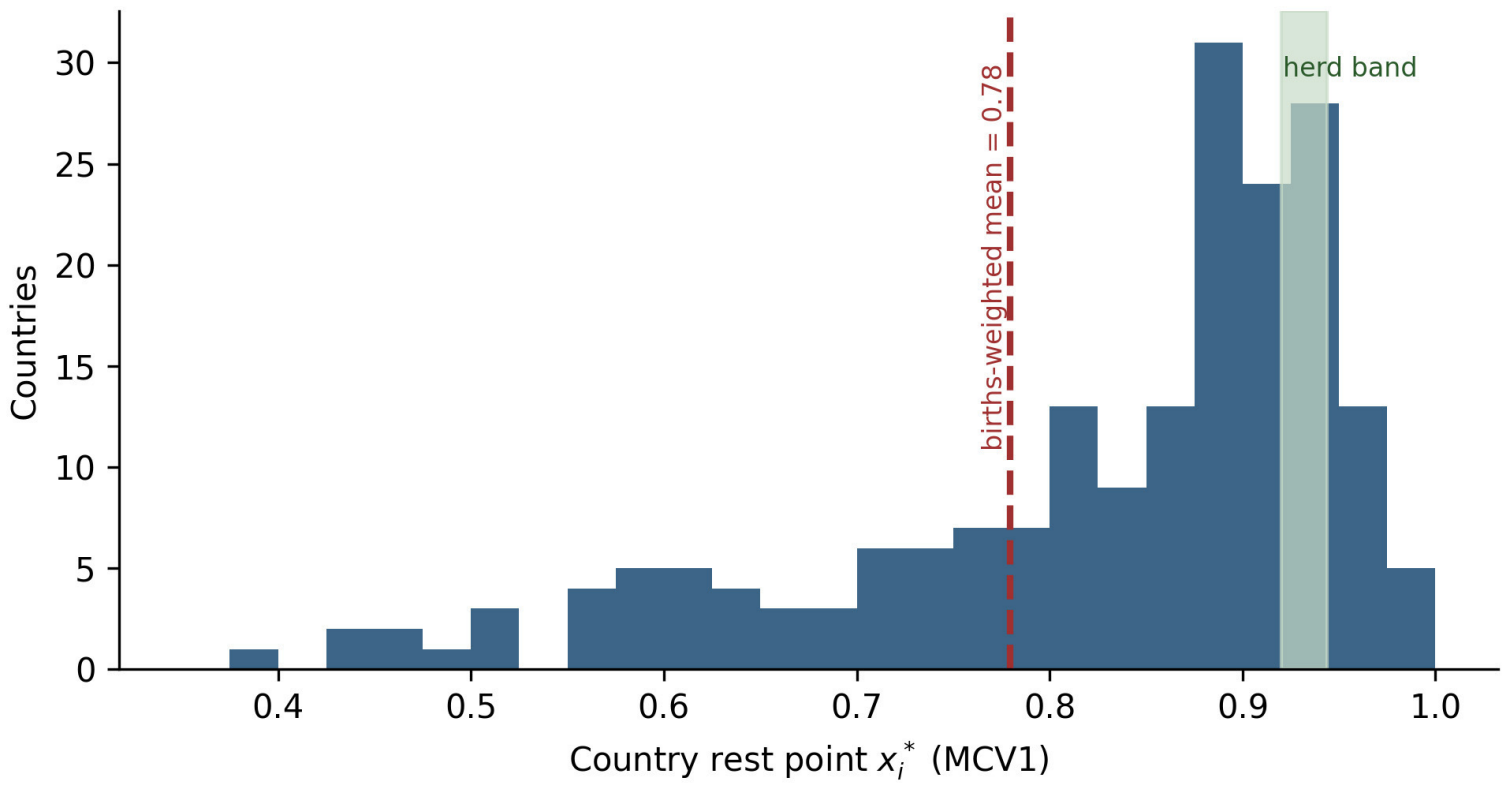
The estimated law of motion of MCV1 coverage. Circles are bin means of the annual change in coverage by lagged coverage (marker area proportional to observations); crosses trace bin means of the fitted values from the two-way fixed-effects specification; the open circle marks the median-country rest point; and the shaded band is the herd immunity band.

**Table 6:** Country rest points x^*^ for MCV1, by income group.

Income group	Countries	Median x^*^	Share with x^*^ < 0.92
Low income	25	0.676	1.00
Lower middle income	47	0.754	0.87
Upper middle income	58	0.878	0.76
High income	63	0.917	0.54
All countries	195	0.876	0.74

Rest points solve the estimated law of motion f(x) = 0 with country-specific intercepts; the births-weighted world mean is 0.780. Income groups follow the World Bank classification carried in the WUENIC file; countries without an income classification in that file appear only in the all-countries row. x^*^ denotes the estimated rest point of the law of motion.

### 3.6 The COVID-era shift

Table 7 interacts the law of motion with an indicator for 2020 onward. The interactions are jointly meaningful: the selection level shifts by +0.659 (*t* = 2.15) and the frequency-dependence term by −0.696 (*t* = −2.26), while the restoring term weakens by +0.136 (*t* = 1.72) and the intercept falls by −0.133 (*t* = −1.70). The individually offsetting signs matter less than the composite: Figure 5 plots the pre-2020 and post-2019 laws of motion for the median country and shows that the post-2019 curve lies below the pre-2020 curve throughout the policy-relevant range, so the stable rest point falls from 0.891 to 0.858, a loss of 3.3 points of steady-state coverage. This is a sharper statement than the familiar observation that coverage dipped during the pandemic (23, 24): the estimates imply that the dip is not a transitory deviation from an unchanged attractor but a displacement of the attractor itself. In the language of Section 2.3, the fall in the intercept is a fall in ρ*q*, the contact and conversion probabilities that the pandemic disrupted directly. The weakening of the restoring term is not evidence of higher attrition: since the restoring coefficient is minus the sum of the enrollment and attrition hazards, a rise in attrition would strengthen it rather than weaken it, and the shift of +0.136 in the restoring coefficient almost exactly offsets the shift of −0.133 in the intercept, so that the value of the law of motion at full coverage, which is minus the attrition hazard by Proposition 5, moves by only 0.003. In this specification, the displacement of the attractor is a contact and conversion shock rather than an attrition shock. Whether a confidence shock of the kind the vaccine-confidence literature documents (22) also operated cannot be read from the mutation terms, which show no rise in attrition; it would have to be sought in the payoff terms, whose post-2019 shifts are individually offsetting. Excluding the pandemic years altogether leaves the baseline turnover estimate essentially unchanged (−0.274, *t* = −10.5 on the 1981–2019 sample), so the headline results are not artifacts of the shock.

**Table 7:** The post-2019 shift in the law of motion (MCV1, country fixed effects).

Dependent variable: Δx	Coefficient	t
Selection level, s (lagged)	0.160	1.81
Frequency dependence, s·x (lagged)	−0.051	−0.45
Linear restoring term, x (lagged)	−0.159	−9.13
Post-2019 × s	0.659	2.15
Post-2019 × s·x	−0.696	−2.26
Post-2019 × x	0.136	1.72
Post-2019 intercept	−0.133	−1.70
Observations	8,019	

Country fixed effects; standard errors clustered by country. Implied median-country rest point: 0.891 before 2020, 0.858 from 2020 onward.

**Figure 5:**
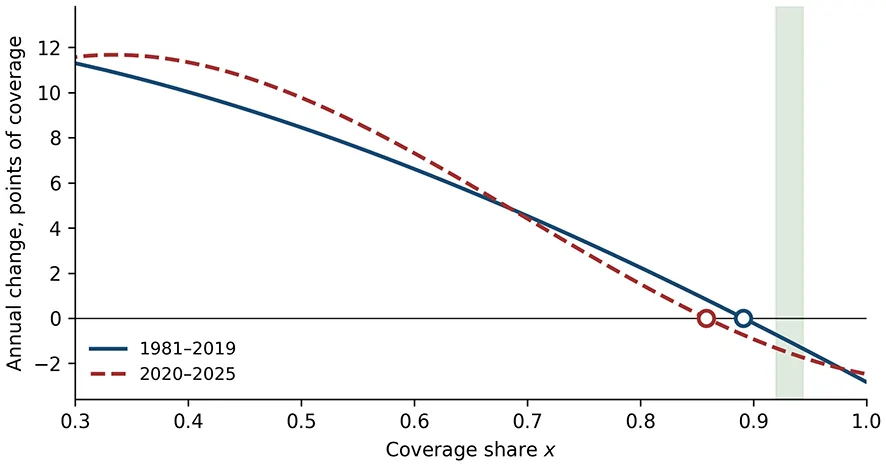
The linear restoring coefficient under alternative dating and series, with 95% confidence intervals. The contemporaneous dating reverses the sign mechanically (division bias); the two-lag dating bounds the behavioral turnover from below; the administrative series doubles it through measurement noise.

### 3.7 Heterogeneity and robustness

The turnover coefficient is remarkably stable across the structural divides of the immunization world: −0.213 in Gavi-supported countries against −0.268 elsewhere, and −0.233 in low- and lower-middle-income countries against −0.266 in upper-middle- and high-income countries, with no significant interior selection terms in any subsample (Supplementary Table S1). What differs across development levels is therefore not the strength of the restoring force but the enrollment hazard that sets where the force comes to rest, namely the income gradient of Table 6. The pooled specification without fixed effects, immune to dynamic-panel bias, retains a strongly negative restoring term (−0.110, *t* = −6.9). With an average time dimension above forty years, the Nickell (52) bias, of order 1/*T*, cannot account for more than a small fraction of the two-way estimate. The dropout margin, defined as the share of the cohort that receives the first but not the third DTP dose, shows strong mean reversion toward a positive interior level, confirming that incomplete schedules are a persistent structural state rather than a transitional one (Supplementary Table S2).

### 3.8 Leverage along the last mile

Table 8 translates Propositions 3 and 4 into the arithmetic implied by the point estimates for the median country. At the median rest point of 0.876, the local slope of the law of motion is ${\hat{f}}^{\prime }\left(x^{*}\right)=-0.344$, which is the two-year half-life of Section 3.5. A uniform increase of 0.01 in the flow payoff raises the rest point by 0.32 coverage points, whereas a reduction of 0.01 in the annual attrition hazard raises it by 2.55 points: the leverage ratio is 8.1, as Equation 5 requires. The cost of reaching a given coverage target through either channel follows directly. Lifting the median country's rest point to 0.90 requires a payoff shift of 0.09 in flow units or an attrition reduction of 0.009; reaching the lower edge of the herd band at 0.92 requires 0.21 or 0.017; reaching 0.94 requires 0.40 or 0.024. The ratio of the two requirements is 1/(1 − *T*) at each target *T*, so it climbs from 10 at 0.90 to 12 and a half at 0.92 and to 17 at 0.94. Table 8 also reports the leverage ratio at the median rest point of each income group: 3.1 in low-income countries, 4.1 in lower-middle-income countries, 8.2 in upper-middle-income countries, and 12.0 in high-income countries. The ordering of instruments is therefore not uniform across the world. Where the rest point is low, the variance factor *x*(1 − *x*) is near its maximum and payoff and contact instruments carry comparable leverage; where the rest point is inside or just below the herd band, retention dominates by an order of magnitude. Section 4.4 reads the intervention literature through Table 8.

**Table 8:** Leverage of retention vs. payoff instruments along the last mile (MCV1, median country).

Quantity	Value
Median rest point x^*^	0.876
Local slope of the law of motion, f′(x^*^)	−0.344
Half-life of convergence (years)	2.0
Effect on x^*^ of a 0.01 uniform payoff shift (coverage points)	0.32
Effect on x^*^ of a 0.01 reduction in attrition hazard (coverage points)	2.55
Leverage ratio 1/(1 − x^*^) at 0.876	8.1
Payoff shift needed to reach 0.90/0.92/0.94 (flow units)	0.09/0.21/0.40
Attrition reduction needed to reach 0.90/0.92/0.94	0.009/0.017/0.024
Ratio of the two requirements at 0.90/0.92/0.94	10.0/12.5/16.7
Leverage ratio at income-group median rest points: low/lower middle/upper middle/high	3.1/4.1/8.2/12.0
Variance factor x(1 − x) at the same rest points	0.219/0.185/0.107/0.076

Computed from the two-way fixed-effects point estimates of Table 3, column S3 with the median-country enrollment hazard of 0.203, using Equations 5 and 7. Payoff shifts are uniform additions to $\sigma \Delta \bar{\pi }$ in annual flow units; attrition reductions are absolute changes in the annual hazard μ_*out*_. Income-group rest points are the medians of Table 6. x^*^ denotes the estimated rest point of the law of motion.

## 4 Discussion

### 4.1 What kind of economic object is national vaccination coverage?

Read together, the results reframe what kind of economic object national vaccination coverage is. The theoretical literature models coverage as the running outcome of a population game in which households chase payoffs that erode with herd protection (5, 7). The estimates indicate that this mechanism is real, visible, and quantitatively strong exactly where it is identified, in rollouts that traverse the simplex, but that in the mature interior where the world's coverage actually sits, the dominant forces are linear in coverage: an institutional enrollment default that recruits about a fifth of the uncovered pool each year and an attrition drift that removes about one in 20 covered households. Mature vaccination coverage is best described not as a Nash equilibrium being continuously recomputed but as a convention (55) that rests where institutional recruitment balances demographic and administrative erosion, with the payoff channel supplying, at the margin, a force throttled by the vanishing variance factor *x*(1 − *x*).

### 4.2 Alternative readings of the linear force

The reframing must be stated with the care that Remark 1 imposes, and three alternative readings of the linear force deserve explicit treatment. The first is latent payoff responsiveness. A linear restoring term is what a best-response protocol produces whenever the probability of choosing vaccination is locally flat in coverage: the revision rate σ itself appears as an outflow proportional to *x*, and a household population that re-optimizes every year but whose choice probability barely moves across the observed window is observationally equivalent, on that window, to a population governed by institutional hazards. Interior data cannot exclude this reading, and I do not claim they can. What the data do exclude is a payoff-responsive process of the imitation form with a large slope in the interior, because that process would generate a significant variance-weighted curvature term, and none appears in any correctly dated interior specification on the reconciled series. What favors the institutional reading over the flat-choice reading is not the interior at all but three pieces of evidence from outside it: the rollout antigens, where the payoff term is significant in exactly the variance-weighted form; the DTP1 margin, where the linear term vanishes precisely on the margin that has no completion component to erode; and the capacity result of column S4, where the drift toward vaccination responds to health-system capacity conditional on income rather than to income conditional on capacity. Each of these is what the institutional reading predicts, and none is what a flat-choice best-response reading would predict without further assumptions. The estimated hazards are nonetheless reported as upper bounds on the institutional component, and the decomposition of turnover into revision and institutional flows is an open question that only microdata on revision behavior can close.

The second alternative reading is measurement. Mean reversion in a smoothed series can masquerade as behavioral turnover, and the WUENIC estimates are the output of a reconciliation rule. Section 3.4 bounds this channel from both sides: the twice-lagged dating, which strips first-order measurement-error contamination, lowers the turnover estimate from 0.25 to 0.15 but does not remove it, and the unsmoothed administrative series, which contains more noise, roughly doubles it. The behavioral component therefore lies within a band whose lower edge is well above zero. The third alternative reading is aggregation. National coverage is a births-weighted average of subpopulations, and a linear national law of motion can arise as the mixture of subpopulation laws with different intercepts even if each subpopulation is individually payoff-responsive, provided the mixture weights are stable. This reading is not an alternative to the institutional one so much as a refinement of it: the subpopulation intercepts are contact and conversion probabilities, and their dispersion is the within-country inequality that Section 4.5 discusses. The national estimates identify the weighted average of these hazards, which is the right object for the aggregate rest point but the wrong object for locating where the uncovered pool sits.

### 4.3 Health systems as the carriers of the institutional flows

The finding that the dominant interior forces are linear in coverage is a statement about health systems, and Table 1 makes the correspondence explicit. The enrollment hazard is the product of a contact probability and a conversion probability. The contact probability is governed by the workforce and service-delivery building blocks: the density of health workers and community health workers, the reach of primary-care facilities and outreach sessions, and the linkage of immunization to birth registration and to other routine contacts. The conversion probability depends on medical products and financing: whether the vaccine is in stock when the child is present, whether the session is held, and whether the default at the point of contact is to vaccinate. Attrition is governed by continuity of care, by the information systems that generate reminders and recall, and by the governance that maintains trust and manages confidence shocks. Recent panel evidence that workforce density, public financing and governance quality are the leading determinants of service coverage in 135 low- and middle-income countries, and that corruption is associated with substantial efficiency losses (18), therefore describes the determinants of ρ, *q* and *d*; the systematic review of effective vaccine coverage determinants reaches the same components from the immunization side (20); and the Lancet Global Health Commission's argument that quality, not access alone, is the binding constraint on health-system performance in the Sustainable Development Goals era (19) is, in the model's terms, an argument about conversion and attrition rather than about contact. The capacity result of Section 3.2 is the empirical counterpart: health expenditure per capita, conditional on income, raises the drift toward vaccination, whereas income conditional on capacity does not. Vaccine stock-outs, which affected a large share of countries in the years for which they have been surveyed (56), are the clearest single example of a conversion failure that no household payoff can offset: a household that has made contact and has decided to vaccinate cannot do so if the vial is absent. The 5As taxonomy of vaccine uptake determinants, access, affordability, awareness, acceptance, and activation (47), maps onto the model in the same way: access and activation are contact and conversion, affordability and acceptance are components of $\bar{c}$ and *L*, and awareness enters both σ and *L*.

This is also where the health-production-function perspective enters. In the tradition of Grossman (38), coverage is an input into child health capital, and its determinants are the prices and the technology that convert household resources into health. The recent decomposition of global healthy-life-expectancy trajectories finds that women's education is associated with immunization coverage more strongly than with any other outcome studied and that wealth-related inequality in DTP3 coverage persists within countries even where national coverage is high (30). The model interprets both findings as statements about the conversion of resources into contact: maternal education raises the probability that a household is in routine contact with the system and acts on that contact, and the within-country wealth gradient is dispersion in the contact and conversion probabilities. Structural barriers that prevent households from converting resources into outcomes, documented in other domains such as the part of the caste gap in dietary diversity in India that differences in endowments do not explain (57), are the general form of the phenomenon: the income coefficient in column S4 is negative conditional on capacity because resources without a system to convert them do not produce coverage.

### 4.4 Policy leverage, cost-effectiveness, and implementation across contexts

This reframing carries a precise policy arithmetic. The classical instruments of the externality literature, Pigouvian subsidies and mandates that shift the payoff difference (2, 4), enter the law of motion multiplied by *x*(1 − *x*), which at the observed rest point equals 0.11; retention instruments enter multiplied by x^*^, so their per-unit leverage exceeds the payoff channel's by 1/(1 − x^*^), about eight at the median rest point and rising without bound along the last mile. Calibrated on the estimates (Table 8), lifting the median country from its rest point of 0.876 into the herd band requires a uniform payoff shift of about 0.21 in flow units but only a 0.017 reduction in the annual attrition hazard, a ratio of about twelve. The practical translation is that reminder and recall systems, default scheduling at birth registration, school-entry verification, provider continuity, and supply reliability, all of which act on μ_*out*_ or μ_*in*_, are not soft complements to price policy; near the herd threshold, they are the high-powered instruments while price-type instruments are the weak ones. This inverts the instrument ranking that a static reading of the externality model suggests, and it is consistent with the observation that the countries closest to the band are those with the strongest routine-contact institutions rather than those with the largest demand-side subsidies. The COVID-era result sharpens the point: the pandemic displaced the attractor itself, so a return to pre-2019 behavior will not occur by relaxation alone and requires an instrument that shifts the rest point back; since Section 3.6 locates the displacement in the contact and conversion probabilities, that instrument lies on the enrollment side.

The intervention literature supports the ordering, and the model explains why its results look contradictory when read without the variance factor. On the retention side, the Cochrane review of patient reminder and recall interventions finds that they raise immunization rates across delivery modes, including low-cost postcards, telephone calls, and text messages (58); a text-message reminder trial in a low-income urban pediatric population raised influenza vaccination significantly through a low-cost delivery channel (59); a cluster-randomized trial in Kenya found that mobile-phone reminders combined with small mobile-money incentives improved the timeliness and completeness of childhood immunization, with the largest incentive producing the largest effect (60); opt-out scheduling of vaccination appointments raised uptake relative to opt-in scheduling with no change in price (61); and states with easier non-medical exemptions from school-entry requirements had higher pertussis incidence, which is the attrition channel operating through the verification default (62). The synthesis of the psychological literature on vaccination concludes that interventions that facilitate action, reminders, defaults, onsite delivery, and requirements have stronger evidence than interventions that aim to change beliefs (63). On the payoff side, the evidence splits by coverage level exactly as *x*(1 − *x*) predicted. In rural Rajasthan, where full immunization stood near 6%, reliable monthly immunization camps raised full coverage to 18%, and camps combined with small in-kind incentives to 39% (64): at *x* = 0.06 the variance factor is 0.056, almost equal to the coverage share itself, so the leverage ratio of retention over payoff instruments is 1.06, and a payoff instrument carries the same per-unit leverage as a retention instrument; the throttle that weakens payoff instruments near the herd band is absent at the low corner, while a contact instrument, whose multiplier is one minus the coverage share, carries more leverage than either, and the reliable camp itself is a ρ*q* intervention. In high-income settings with coverage in the 0.85 to 0.95 range, by contrast, the systematic review of parental financial incentives for preschool vaccination finds only a small and methodologically limited body of evidence that incentives raise uptake (65), which is what a leverage ratio of 7 to 20 in favor of retention instruments implies. The model does not say that incentives fail; it says that their leverage is a function of where the country sits on the simplex, and Table 8 gives that function.

Cost-effectiveness and implementation follow the same gradient. Childhood immunization is among the highest-return health investments in low- and middle-income countries (66), so the relevant question is not whether to spend but on which parameter. In the low-income group, where the median rest point is 0.676 and every country rests below the band, the binding constraint is contact: the zero-dose children who never enter the immunization cascade are concentrated in the poorest households and the most remote areas (28, 29), and the instruments with leverage are outreach sessions, community health workers, reliable supply, and, because the variance factor is large, demand-side incentives as well. The Cochrane review of interventions in low- and middle-income countries finds that facility- and community-based health education, home visits, and combinations of these with incentives raise coverage (67). In the high-income group, where the median rest point is 0.917 and the leverage ratio is twelve, the binding constraint is retention and confidence: reminder and recall systems, default scheduling, provider continuity, and school-entry verification are the instruments with leverage, and the last three to five points of coverage will not be bought with price instruments. Between these poles, the upper-middle-income group with a median rest point of 0.878 is the case where the leverage ratio has already reached eight and the transition from contact-side to retention-side instruments is due. Implementation therefore differs by context not in kind but in the parameter targeted, and the model gives a single rule for choosing it: locate the country's rest point, read the leverage ratio, and spend on the channel with the larger derivative.

### 4.5 Gender, intra-household decisions, and intersecting inequalities

The aggregate panel is silent about who inside the household decides, but the model is not, and the gender evidence can be read through it. In most settings the caregiver who bears the time cost of a completed schedule is the mother, and her mobility, autonomy, education, and time budget determine three of the model's parameters at once: the time component *w*_*i*_τ_*i*_ of the cost, the contact probability ρ, and the attrition component *m* through migration and household disruption (32, 33). The meta-ethnographic review of gender determinants of child vaccination finds that women's low decision-making autonomy, restricted mobility, and competing time demands are recurrent barriers (33); read through the model, these barriers act at the point of contact rather than at the point of valuation, since a mother who values vaccination may still be unable to reach the session or to decide alone to attend, which is a low ρ and a high *w*_*i*_τ_*i*_ rather than a low *L*. Women's time poverty is the quantitative face of this constraint: where employed women perform several times more unpaid work than men and total workloads approach 10 h a day, as the 2024 Indian time-use survey documents, the shadow value of the hours a schedule requires is high and rises with the distance to the facility (68). The collective-household literature adds the bargaining dimension: when the decision rests with a member other than the caregiver, the caregiver's information and time cost are discounted, and resources controlled by women are more likely to be spent on children's health (41, 42). Gender norms therefore enter the law of motion twice, through $\bar{c}$ in the payoff channel and through ρ in the mutation channel, and the article's decomposition says which entry matters more in the interior: since the payoff channel is throttled by *x*(1 − *x*), the contact entry dominates. This is consistent with the ecological evidence that subnational gender inequality correlates with immunization coverage and that women's education is more strongly associated with DTP3 coverage than with other outcomes (30, 31): these are associations between women's position and the probability that the household is reached and acts on being reached.

Gender intersects with wealth, residence, and social group, and the intersection matters for the herd-immunity question in a specific way. Let the population consist of groups *g* with birth shares *w*_*g*_ and group-specific hazards μ_*in,g*_ and μ_*out,g*_; each group has its own rest point $x_{g}^{*}$, and the national rest point is $\underset{g}{\sum }w_{g}x_{g}^{*}$. Herd protection, however, is a property of who mixes with whom. With assortative mixing, the effective reproduction number depends on coverage within groups, so the threshold must be met inside each group, and a national rest point inside the band is compatible with groups far below it (25); clustering of unvaccinated households raises outbreak probability at any given national coverage (26). The persistent wealth gradient in full immunization and the concentration of zero-dose children among the poorest households (27–29), and the finding that DTP3 inequality remains substantial in countries with high national coverage (30), are therefore not footnotes to the aggregate result but the mechanism through which a country that appears to sit near the band still experiences outbreaks. The policy implication follows from Proposition 3 applied group by group: because the leverage ratio depends on the group's own rest point, retention instruments dominate in the high-coverage groups while contact instruments and incentives retain their leverage in the low-coverage groups, and a national program that spends uniformly across groups spends in the wrong proportions in both. The national panel cannot estimate the group-specific hazards; household survey data with wealth, residence, and caregiver characteristics can, and estimating the law of motion on such data is the extension that would make the gender and intersectional channels quantitative rather than interpretive.

### 4.6 Measurement policy

The findings also speak to measurement policy. The same estimates that reveal the turnover channel show how easily it is mismeasured: contemporaneous dating reverses the sign of the central coefficient, and replacing the reconciled WUENIC series with raw administrative coverage doubles the apparent turnover. Analysts estimating behavioral dynamics on coverage statistics should treat timing conventions and the provenance of the series as first-order design choices, not as robustness afterthoughts.

### 4.7 Limitations

Several limitations bound the claims. First, the analysis is conducted on national aggregates; the household-level decision model is a microfoundation for the aggregate law of motion, not a claim estimated on microdata, and subnational heterogeneity in coverage, which matters greatly for outbreak risk, is invisible at this resolution; Section 4.5 states what the aggregate estimates can and cannot say about it. Second, the interior weak identification of the frequency-dependence slope is a property of the data's support, not of the estimator, and it limits what any aggregate panel can say about free riding in the mature range; the rollout-based identification is the honest substitute, and it is external to the interior in exactly the way instrument-based designs usually are. Third, the identification of the institutional hazards is parametric and protocol-specific: it relies on the affine payoff function and on the imitation protocol, and under a best-response protocol the recovered hazards are upper bounds that include payoff-insensitive revision. Testing the affine form against higher-order terms and estimating the revision rate from microdata on switching behavior are the two exercises that would tighten these bounds. Fourth, the outbreak-responsiveness channel, through which local cases raise perceived risk and feed back into uptake (69), could not be estimated here because the reported-case extract available to this study is the global aggregate; country-level surveillance panels would permit that extension and are the natural next step. Fifth, the enrollment and attrition hazards are behavioral-institutional composites; decomposing them into supply reliability, demographic churn, and confidence shocks requires program-level data, and the health-system mapping of Table 1 is a framework for that decomposition rather than an estimate of it. Sixth, WUENIC itself embodies estimation rules that change across revisions, and although the administrative-series and grade-restricted checks bound the resulting measurement effects, they cannot eliminate them. Seventh, the policy leverage result is a comparison of derivatives at the rest point; it orders instruments per unit of parameter shift, and converting that ordering into a cost-effectiveness ranking requires the cost of moving each parameter by one unit in a given setting, which the intervention literature supplies only partially and only for some settings.

## 5 Conclusion

This study estimated, for the first time on the global immunization panel, the law of motion that health economics has long asserted for vaccination coverage, derived it from household choice through an explicit revision process, and showed exactly which of its parameters the data identify. The free-riding mechanism at the heart of the externality argument is present and strong where the data can identify it, in national rollouts that traverse the whole coverage range, but the day-to-day dynamics of mature coverage are governed by flows that are linear in coverage, an enrollment default of roughly 0.20 and an attrition drift of roughly 0.045 per year, whose balance pins every country at a stable interior rest point reached with a 2-year half-life. These flows are health-system quantities, the product of contact and conversion probabilities and the sum of churn, discontinuity, and confidence losses, and they bound the institutional component of turnover from above. The world's births-weighted rest point for first-dose measles coverage is 78%, 14 points below the herd band, and the pandemic shifted the median country's rest point down by a further 3.3 points. Because the leverage of retention instruments over payoff instruments grows as 1/(1 − x^*^) along the last mile under the imitation protocol and diverges along it under any smooth, bounded choice protocol, the policy conclusion is structural rather than rhetorical: herd immunity will not be priced into existence; it must be defaulted and retained into existence. Estimating the same law on subnational, survey-based, and surveillance-linked panels, where gender, wealth, and residence can be observed, and decomposing the institutional hazards into their supply and confidence components are the immediate extensions.

## Data Availability

Publicly available datasets were analyzed in this study. The WHO/UNICEF Estimates of National Immunization Coverage (WUENIC, 2025 revision) and the WUENIC production input file are available at https://immunizationdata.who.int; the United Nations World Population Prospects 2024 at https://population.un.org/wpp; and the World Bank World Development Indicators at https://databank.worldbank.org. Further inquiries can be directed to the corresponding author.

## Supplementary material

Supplementary Table S1 The law of motion of MCV1 coverage by Gavi-support status and by income group.

Supplementary Table S2 The law of motion of the dropout margin between the first and third DTP doses.
