# Supplementary material for: The herd immunity attractor: selection, drift, and the steady-state undersupply of vaccination in 195 countries, 1980–2025
Source: Front Public Health. 2026 Oct 22;14:1965676. doi: 10.3389/fpubh.2026.1965676 (PMC13735566; doi:10.3389/fpubh.2026.1965676)
Supplement: Supplementary file 1 [file Table_1.docx]

# Supplementary Material

## The Herd Immunity Attractor: Selection, Drift, and the Steady-State Undersupply of Vaccination in 195 Countries, 1980–2025

Aras Yolusever^1*^

^1^ Department of Economics, Faculty of Economics and Administrative Sciences, Istanbul Kültür University, Istanbul, Türkiye

* Correspondence: Aras Yolusever, a.yolusever@iku.edu.tr

## Supplementary Tables

**Supplementary Table S1. Heterogeneity of the law of motion (MCV1, two-way fixed effects).**

| Dependent variable: Δx | Gavi-supported | Non-Gavi | Low and lower-middle income | Upper-middle and high income |
| --- | --- | --- | --- | --- |
| Selection level, s (lagged) | 0.177 (1.53) | 0.154 (0.96) | 0.140 (1.27) | 0.124 (0.66) |
| Frequency dependence, s·x (lagged) | −0.143 (−0.84) | −0.055 (−0.32) | −0.040 (−0.25) | −0.073 (−0.36) |
| Linear restoring term, x (lagged) | −0.213 (−6.60) | −0.268 (−7.32) | −0.233 (−7.48) | −0.266 (−6.27) |
| Observations | 2,348 | 5,671 | 2,962 | 4,970 |

t statistics in parentheses, standard errors clustered by country. Gavi support follows the Gavi57 classification carried in the WUENIC file; income groups follow the World Bank classification.

**Supplementary Table S2. Additional robustness (MCV1).**

| Dependent variable: Δx (Δd in the last column) | Pooled, no fixed effects | Pre-2020 sample (1981–2019) | Dropout margin d = DTP1 − DTP3 |
| --- | --- | --- | --- |
| Selection level, s (lagged) | 0.031 (0.39) | 0.090 (0.92) | 0.672 (1.15) |
| Frequency dependence, s·x (lagged) | −0.063 (−0.64) | 0.087 (0.71) | 2.087 (2.32) |
| Linear restoring term, x (lagged) | −0.110 (−6.90) | −0.274 (−10.54) | −1.210 (−1.99) |
| Country fixed effects | no | yes | yes |
| Year fixed effects | no | yes | yes |
| Observations | 8,019 | 6,849 | 8,144 |

t statistics in parentheses, standard errors clustered by country. The pooled specification is free of dynamic-panel bias; the pre-2020 sample excludes the pandemic years; the dropout margin applies the estimating equation to the share of the cohort receiving the first but not the third DTP dose, whose own lagged level and variance terms replace those of coverage.
